# Chitinase-3-like 1 protein (*CHI3L1*) locus influences cerebrospinal fluid levels of YKL-40

**DOI:** 10.1186/s12883-016-0742-9

**Published:** 2016-11-10

**Authors:** Yuetiva Deming, Kathleen Black, David Carrell, Yefei Cai, Jorge L. Del-Aguila, Maria Victoria Fernandez, John Budde, ShengMei Ma, Benjamin Saef, Bill Howells, Sarah Bertelsen, Kuan-lin Huang, Courtney L. Sutphen, Rawan Tarawneh, Anne M. Fagan, David M. Holtzman, John C. Morris, Alison M. Goate, Joseph D. Dougherty, Carlos Cruchaga

**Affiliations:** 1 Department of Psychiatry, Washington University School of Medicine, 660 S. Euclid Ave. B8134, St. Louis, MO 63110 USA; 2 Ronald M. Loeb Center for Alzheimer’s disease, Department of Neuroscience, Icahn School of Medicine at Mount Sinai, New York, NY USA; 3 Department of Genetics, Washington University School of Medicine, 660 S. Euclid Ave., St. Louis, MO 63110 USA; 4 Department of Neurology, Washington University School of Medicine, 660 S. Euclid Ave., St. Louis, MO 63110 USA; 5 Knight Alzheimer’s Disease Research Center, Washington University School of Medicine, 660 S. Euclid Ave., St. Louis, MO 63110 USA; 6 Hope Center for Neurological Disorders, Washington University School of Medicine, 660 S. Euclid Ave. B8111, St. Louis, MO 63110 USA; 7 Department of Developmental Biology, Washington University School of Medicine, 660 S. Euclid Ave., St. Louis, MO 63110 USA

**Keywords:** CHI3L1, YKL-40, Cerebrospinal fluid, Alzheimer disease

## Abstract

**Background:**

Alzheimer’s disease (AD) pathology appears several years before clinical symptoms, so identifying ways to detect individuals in the preclinical stage is imperative. The cerebrospinal fluid (CSF) Tau/Aβ_42_ ratio is currently the best known predictor of AD status and cognitive decline, and the ratio of CSF levels of chitinase-3-like 1 protein (*CHI3L1*, YKL-40) and amyloid beta (Aβ_42_) were reported as predictive, but individual variability and group overlap inhibits their utility for individual diagnosis making it necessary to find ways to improve sensitivity of these biomarkers.

**Methods:**

We used linear regression to identify genetic loci associated with CSF YKL-40 levels in 379 individuals (80 cognitively impaired and 299 cognitively normal) from the Charles F and Joanne Knight Alzheimer’s Disease Research Center. We tested correlations between YKL-40 and CSF Tau/Aβ_42_ ratio, Aβ_42_, tau, and phosphorylated tau (ptau_181_). We used studentized residuals from a linear regression model of the log-transformed, standardized protein levels and the additive reference allele counts from the most significant locus to adjust YKL-40 values and tested the differences in correlations with CSF Tau/Aβ_42_ ratio, Aβ_42_, tau, and ptau_181_.

**Results:**

We found that genetic variants on the *CH13L1* locus were significantly associated with CSF YKL-40 levels, but not AD risk, age at onset, or disease progression. The most significant variant is a reported expression quantitative trait locus for *CHI3L1*, the gene which encodes YKL-40, and explained 12.74 % of the variance in CSF YKL-40 in our study. YKL-40 was positively correlated with ptau_181_ (r = 0.521) and the strength of the correlation significantly increased with the addition of genetic information (r = 0.573, *p* = 0.006).

**Conclusions:**

CSF YKL-40 levels are likely a biomarker for AD, but we found no evidence that they are an AD endophenotype. YKL-40 levels are highly regulated by genetic variation, and by including genetic information the strength of the correlation between YKL-40 and ptau_181_ levels is significantly improved. Our results suggest that studies of potential biomarkers may benefit from including genetic information.

**Electronic supplementary material:**

The online version of this article (doi:10.1186/s12883-016-0742-9) contains supplementary material, which is available to authorized users.

## Background

Since it has been well demonstrated that Alzheimer’s disease (AD) pathology is present long before any clinical symptoms, emphasis has been on improving identification of those in this preclinical stage [1–3]. Studies have found that the ratio of cerebrospinal fluid (CSF) tau to Aβ_42_ is a better predictor of AD status and cognitive decline than either of these proteins individually and improves discrimination of AD from other dementias such as vascular dementia and frontotemporal lobar degeneration [4–6]. CSF levels of tau correlate with the amount of neurodegeneration, phosphorylated tau (ptau) levels correlate with tangle pathology, and Aβ_42_ levels inversely correlate with the amount of plaques, which makes these ideal biomarkers for AD pathology [3, 7]. Studies have focused on targeting Aβ, tau, or both proteins as potential treatments for AD, making it necessary to find alternative biomarkers that are not being directly targeted [8–11]. CSF levels of YKL-40 are a promising biomarker for AD; they are significantly higher in individuals with AD dementia than in cognitively normal individuals [12]. CSF YKL-40 is also associated with cortical thinning in cognitively normal individuals with low levels of CSF Aβ_42_ who are at risk for AD, and highly correlates with CSF ptau [13]. YKL-40 is a secreted glycoprotein, encoded by chitinase-3-like 1 protein (*CHI3L1*), expressed in astrocytes and associated with neuroinflammatory response. Although the exact function of YKL-40 is unknown, it has been associated with many immune and inflammatory diseases as well as several cancers [14, 15]. Studies have shown that CSF levels of YKL-40 can be used to distinguish AD from non-AD dementia, Parkinson’s disease, dementia with Lewy bodies, and to distinguish between mild cognitive impairment (MCI) that progresses to AD vs MCI not due to AD indicating that CSF YKL-40 is not simply a marker for inflammation due to neurodegeneration but may be specific enough for AD [16, 17]. However, due to individual variability and group overlap in these CSF protein levels, further studies are necessary to find ways to improve sensitivity and specificity for AD. We hypothesized that adding genetic information may provide a means to improve specificity and sensitivity of CSF YKL-40 as a biomarker for AD.

One key feature of endophenotypes is that they have a clear genetic connection with the trait of interest, and for a biomarker to be considered an endophenotype it has to be measureable, heritable, and segregate with status. The genetic connection between endophenotypes and complex traits has been demonstrated to provide power in genetic studies to identify novel variants and inform about possible underlying biology [18, 19]. CSF levels of tau, ptau_181_, Aβ_42_ are well-established AD endophenotypes and have allowed us to identify novel variants associated not only with tau and ptau_181_ levels but also with AD risk, tangle pathology, and cognitive decline [18]. Recent research suggests that neuroinflammation is not merely a by-product of neurodegeneration in AD but may play a key role in pathology [20]. Since CSF levels of YKL-40 can distinguish between AD and non-AD MCI, perhaps the unknown function of YKL-40 in neuroinflammation is involved in the progression of pathology. If polymorphisms regulating YKL-40 also contribute to some aspect of AD such as risk, age at onset, or cognitive decline then understanding their genetic regulation can help provide insights into biological mechanisms underlying AD, and can help to determine whether YKL-40 is really involved in the pathogenesis (endophenotype) or is just a biomarker for the disease.

First we performed single variant genetic analyses of CSF levels of YKL-40 to identify single nucleotide polymorphisms (SNPs) associated with YKL-40 levels. Next we analyzed whether genome-wide significant variants from our analyses were also associated with AD risk, age at onset, or progression. Finally, we tested correlations between YKL-40 and the CSF Tau/Aβ_42_ ratio, Aβ_42_, tau, and ptau_181_, before and after including genetic information for YKL-40 levels, to determine if adding genetic information can improve the correlation with other known biomarkers, especially ptau_181_.

## Methods

### Ethics statement

The Washington University Institutional Review Board approved this study. Written informed consent was obtained from participants or their family members.

### Study participants

All 379 individuals with measured CSF levels of YKL-40 were from the Charles F. and Joanne Knight Alzheimer’s Disease Research Center (Knight-ADRC; Table 1). Dementia severity was determined using the Clinical Dementia Rating (CDR) where 0 indicates cognitive normality, 0.5 is defined as very mild dementia, 1 is mild dementia, 2 is moderate dementia, and 3 is severe dementia [21]. There were 80 cases, defined as individuals with CDR > 0 at lumbar puncture, and 299 cognitively normal controls (CDR = 0 at lumbar puncture; Table 1). Neuropsychological and clinical assessments were collected for all participants and CSF was collected in the morning after an overnight fast, processed, and stored at -80 °C, as described previously [22]. Individuals were evaluated by Clinical Core personnel at Washington University.

**Table 1:** Characteristics of CSF YKL-40 data

	Knight-ADRC
Samples	379
Age in years (mean ± SD)	70.86 ± 8.80
(range)	49–91
Gender (M/F)	148/231
% CDR >0	21.11
% APOE E4	36.68
CSF YKL-40 (ng/mL)	307.25 ± 108.45

### Genotyping and quality control

The samples were genotyped with the Illumina 610 or Omniexpress chip. Stringent quality control criteria were applied to each genotyping array separately before combining data. A ≤98 % call rate was applied for single nucleotide polymorphisms (SNPs) and individuals. SNPs not in Hardy-Weinberg equilibrium (*p* < 1 × 10^-6^) or with MAF <0.02 were excluded. X-chromosome SNPs were analyzed to verify gender identification. Pairwise genome-wide estimates of proportion identity-by-descent were used to find duplicate and related individuals which were eliminated from the analysis. Principal components were calculated using EIGENSTRAT [23] to confirm ethnicity of each sample. Imputation was performed as described previously [18]. Briefly, BEAGLE v3.3.1 software [24] and the 1,000 genome data were used to impute up to 6 million SNPs. There were 5,986,883 imputed and genotyped SNPs after removing SNPs with a call rate <95 % or a BEAGLE r^2^ ≤ 0.3.

### Analyte measurements and quality control

The Knight-ADRC Biomarker Core measured CSF levels of Aβ_42_, tau, and ptau_181_ using single-analyte enzyme-linked immunosorbent assays (ELISA) as described previously [22]. CSF YKL-40 levels were measured using the MicroVue ELISA (Quidel) as described previously [12].

### Statistical analyses

Log-transformed, standardized values for CSF YKL-40 levels were tested for normality using the Shapiro-Wilk test. We used R v3.2.1 [25] to perform linear regression to determine if CSF levels of YKL-40 were influenced by age, gender, or sample batch (Additional file 1: Table S1). Age, gender, and sample batch were used as covariates to test association of YKL-40 with cognitive status. Correlations of CSF levels of YKL-40 with CSF Aβ_42_, tau, ptau_181_, and Tau/Aβ_42_ ratio were calculated using Pearson’s correlation.

Genetic association with CSF levels of YKL-40 were tested using an additive model in PLINK v1.9 (http://www.cog-genomics.org/plink2) [26]. Covariates used were sample batch, age, gender, and two principal component factors for population structure. Statistical significance for single variant association was defined as *p* < 5 × 10^-8^ based on the commonly used threshold considered appropriate for the likely number of independent tests with Bonferroni correction. The threshold of *p* < 1 × 10^-5^ was defined for suggestive association. The genomic inflation factor for association with YKL-40 levels was 1, suggesting no evidence of inflation due to population stratification. SNP annotation was performed using ANNOVAR version 2015-06-17 [27] and the NCBI Database of Single Nucleotide Polymorphisms (dbSNP) Build ID: 142 (http://www.ncbi.nlm.nih.gov/SNP) [28]. RegulomeDB v1.1 (http://regulome.stanford.edu/index) [29] was used to determine if SNPs of interest were potential regulatory elements. The Genotype-Tissue Expression (GTEx) Analysis Release V6, dbGaP Accession phs000424.v6.p1 (http://www.gtexportal.org) [30] was used to determine if SNPs of interest were potential expression quantitative trait loci (eQTLs).

Disease progression was modeled as the change in CDR Sum of Boxes (CDR-SB) per year. A total of 1,646 individuals from longitudinal studies of AD patients with ≥3 clinical assessments over 1.5 years after being diagnosed with AD were included in the analysis. A mixed-model repeated measure framework was used to account for correlation between repeated measures in the same individual. Age, sex, baseline CDR, follow-up time, level of education, site, and PCs were included as covariates. The appropriate optimal variance-covariance structure that minimizes the Akaike Information Criterion for testing the null model AR1 was selected [31].

To estimate the proportion of variance in CSF levels of YKL-40 explained by genetic variants we used the coefficient of determination (R^2^) of a linear model of the log-transformed, standardized CSF protein levels and the additive reference allele counts of the top genome-wide associated SNP (rs10399931) with sample batch, age, and gender as covariates and subtracted the R^2^ of a linear model with the log-transformed, standardized CSF protein levels and the covariates in the null model. To adjust CSF levels of YKL-40 for genetic effect, we used studentized residuals from a linear regression model of the log-transformed, standardized protein levels and the additive reference allele counts of rs10399931. Age, gender, and sample batch were used as covariates to test association of the adjusted levels of YKL-40 with AD status. Correlations of the adjusted levels of YKL-40 with CSF Aβ_42_, tau, ptau_181_, and tau/Aβ_42_ ratio were determined using Pearson’s correlation (r). The R package cocor version 1.1-1 [32] was used to compare correlations between CSF Aβ_42_, tau, ptau_181_, and tau/Aβ_42_ ratio and adjusted or unadjusted CSF protein levels. We reported the results using the Meng Z-test model [33], but the results for all models reported by cocor were comparable.

See Additional file 2: Supplementary Methods and Results for gene ontology over-representation and tissue-specific expression analyses.

## Results

### CSF levels of YKL-40 in AD cases vs controls

After applying stringent quality control, our dataset contained CSF levels of YKL-40 for 379 individuals from the Charles F. and Joanne Knight Alzheimer’s Disease Research Center (Knight-ADRC; Table 1). We used logistic regression including age, gender, and sample batch as covariates to test whether levels of YKL-40 were associated with AD status defined by CDR. Cases (defined by CDR at lumbar puncture >0) had significantly higher CSF levels of YKL-40 than controls (cases: 366.37 ± 136.48 ng/mL; controls: 290.18 ± 92.44 ng/mL; *p* = 0.015, β = 0.698; Additional file 3: Figure S1). We used linear regression to test if *APOE* genotype influenced YKL-40 and found there was no effect on YKL-40 levels (*p* = 0.704). Several studies indicate that CSF Tau/Aβ_42_ ratio is a better predictor of disease status than clinical assessment [5, 6]. We tested the correlation between CSF Tau/Aβ_42_ ratio and YKL-40. Levels of YKL-40 were positively correlated with CSF Tau/Aβ_42_ ratio (*p* = 2.61 × 10^-8^, r = 0.318), most likely due to the positive correlation with tau (*p* = 7.06 × 10^-22^, r = 0.522) since tau and Tau/Aβ_42_ ratio are highly correlated (*p* = 3.99 × 10^-52^, r = 0.736) and although there is a high negative correlation between Tau/Aβ_42_ ratio and Aβ_42_ levels (*p* = 3.07 × 10^-64^, r = -0.788), YKL-40 was not correlated with Aβ_42_ (*p* = 0.838, r = 0.012; Table 2 and Additional file 4: Figure S2).

**Table 2:** Correlations of CSF levels of YKL-40 with CSF Tau/Aβ ratio, AB42, ptau_181_, and tau levels before and after adjusting for top SNP effect

	CSF Tau/Aβ ratio		CSF Aβ_42_ levels		CSF ptau_181_ levels		CSF tau levels	
	*p*	r	*p*	r	*p*	r	*p*	r
CSF YKL-40	2.61×10^-8^	0.318	0.838	0.012	8.98×10^-22^	0.521	7.06×10^-22^	0.522
CSF YKL-40-rs10399931 effect	5.28×10^-10^	0.357	0.972	0.002	2.82×10^-26^	0.573	1.66×10^-26^	0.575
	*p*	95 % CI	*p*	95 % CI	*p*	95 % CI	*p*	95 % CI
Correlation difference (Meng Z-test)	0.071	-0.083, 0.003	0.661	-0.031, 0.049	0.006	-0.116, -0.020	0.006	-0.116, -0.020

95 % CI: 95 % confidence interval, null hypothesis was retained if interval included 0

### Single variant analysis of CSF YKL-40 levels

**Fig. 1:**
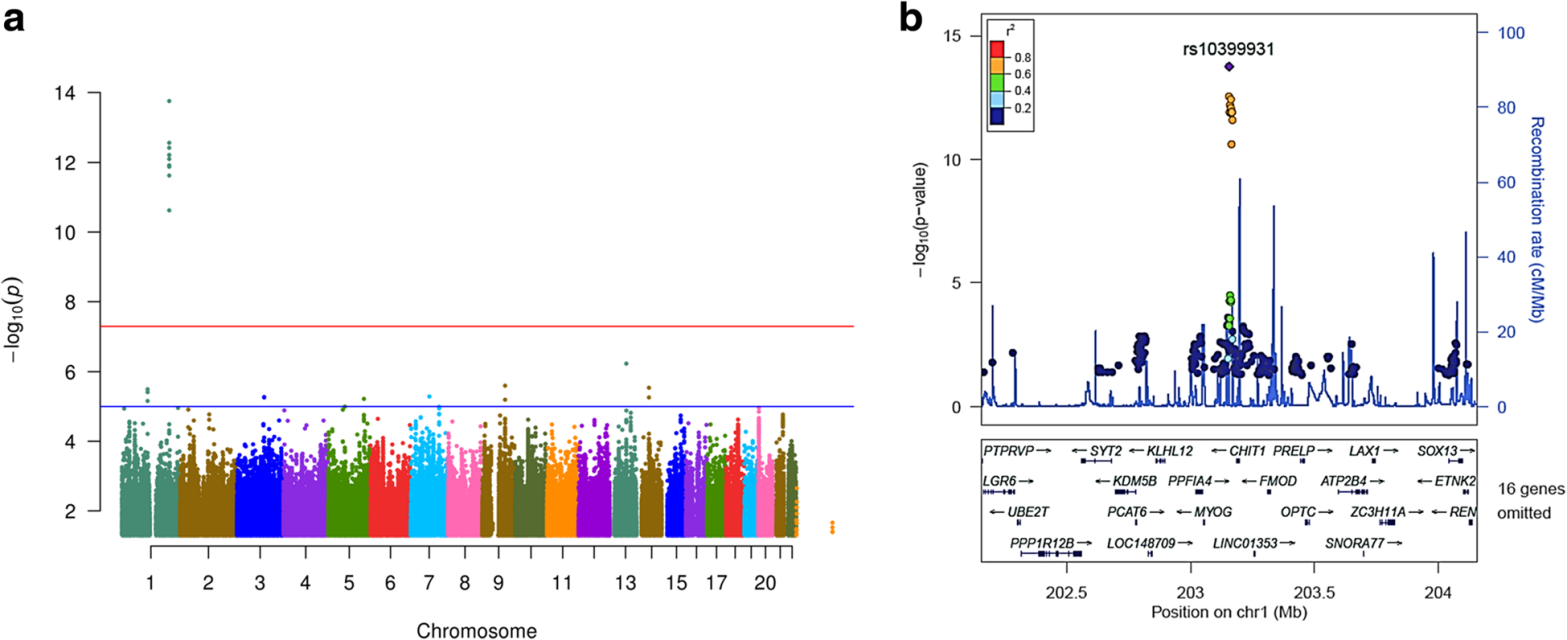
Manhattan and regional plots for associations with CSF levels of YKL-40. **a** Manhattan plot of –log_10_ p-values for genetic association with CSF levels of YKL-40; **b** Regional plot for genome-wide significant association on chromosome 1

To determine whether genetic variants are associated with levels of YKL-40, we used linear regression to test the additive genetic model of each single nucleotide polymorphism (SNP) for association with CSF protein levels using age, gender, sample batch, and two principal component factors for population stratification as covariates. We found 14 genome-wide significant SNPs associated with YKL-40, all in the *CHI3L1* locus. The most significant SNP was rs10399931, a variant <200 bp upstream of the transcription start site for *CHI3L1* (genotyped, *p* = 1.76 × 10^-14^, β = -0.575, minor allele frequency (MAF) = 0.244; Fig. 1 and Additional file 5: Table S2). We did not find any additional genome-wide significant loci when we ran the analysis conditioned on rs10399931. Data from RegulomeDB [29] indicates that rs10399931 is likely to affect binding and gene expression; two RNA-Seq studies and the Genotype-Tissue Expression (GTEx) database have reported rs10399931 is an eQTL for *CHI3L1* (whole blood: *p* = 1.7 × 10^-11^, β = -0.35; transformed fibroblasts: *p* = 3.1 × 10^-11^, β = -0.37; thyroid: *p* = 5.7 × 10^-10^, β = -0.42; lung: *p* = 6.7 × 10^-9^, β = -0.37; tibial nerve: *p* = 8.1 × 10^-9^, β = -0.33; subcutaneous adipose: *p* = 5.5 × 10^-7^, β = -0.28; tibial artery: *p* = 2.4 × 10^-6^, β = -0.26) [30, 34, 35]. *CHI3L1* variants have been associated with YKL-40 levels in serum previously [36], but to our knowledge this is the first time it has been reported to be associated with CSF levels of YKL-40. We calculated what proportion of variance in CSF YKL-40 levels was explained by rs10399931. Age, gender, and sample batch explained 14.89 % of the variance in YKL-40 levels, and addition of the rs10399931 effect explained another 12.74 % of the variance. As a comparison, a previous GWAS study of CSF levels of tau and ptau_181_ estimated that the genetic effect of the genome-wide significant association located in apolipoprotein E (*APOE*) explained only 0.25–0.29 % of the variability of tau and ptau_181_ levels [18]. Additionally, we found a signal in chromosome 13 (rs78081700, *p* = 6.26 × 10^-8^, β = 0.636, Fig. 1 and Additional file 5: Table S2) that almost reached genome-wide significance and another eight loci with suggestive *p* values (Fig. 1 and Additional file 5: Table S2), indicating that additional loci/genes could be associated with CSF YKL-40, although a larger sample size would be needed to confirm this hypothesis.

### Association of GWAS hits and pathways with AD risk, age at onset, and disease progression

To determine if our genome-wide significant SNPs were also associated with risk for AD we searched the results from a GWAS previously published by the International Genomics of Alzheimer’s Project (I-GAP) consisting of a total 25,580 AD cases and 48,466 controls [37]. None of our genome-wide significant SNPs had significant *p*-values in the I-GAP results (rs10399931: *p* = 0.763, β = 0.006; Table 3). Based on analyses of data from a previously published GWAS investigating genetic variants associated with age at onset for AD [38] our genome-wide significant SNPs did not appear to be associated with age at onset (rs10399931: *p* = 0.197, β = 0.026; Table 3). We also found that our genome-wide significant SNPs were not significantly associated with advancement in CDR (rs10399931: *p* = 0.142, β = -0.072; Table 3). Recently the I-GAP published results from a study of biological pathways and gene expression networks associated with AD [39]. None of the gene ontology terms that were enriched in our gene ontology analyses of GWAS results for YKL-40 (Additional file 6: Table S3) were found in the I-GAP report [39]. Together these data suggest that YKL-40 may be a promising biomarker for AD, but probably not an endophenotype.

**Table 3:** Association of genome-wide significant locus from CSF YKL-40 GWAS with AD risk, age at onset, and disease progression

					CSF YKL-40		AD risk		Age at onset		Progression	
Chr	Position	SNP	A1	MAF	*p*	β	*p*	β	*p*	β	*p*	β
1	203156080	rs10399931	T	0.244	1.76×10^-14^	-0.575	0.763	0.006	0.197	0.026	0.142	-0.072

### Improving CSF biomarkers and CSF YKL-40 utility by including genetic information

Because the genome-wide significant SNPs are not associated with disease status but explain a large proportion of the CSF levels of YKL-40, we hypothesized that by accounting for genetic information, it would be possible to improve the efficacy of these measures as biomarkers. Levels of YKL-40 have been reported to be correlated with CSF levels of tau and ptau_181_ but not Aβ_42_, and this was replicated in our analyses (Table 2 and Additional file 4: Figure S2) [12, 15].

We decided to focus on CSF ptau_181_ and YKL-40 since these were highly correlated and the results were similar for tau. First we used linear regression to determine whether rs10399931 genotype influenced ptau_181_ levels (*p* = 0.782, R^2^ = -0.005). Then we included the additive model for rs10399931 in YKL-40 levels and re-analyzed the correlation with ptau_181_. The correlation coefficient for the adjusted values of CSF YKL-40 levels and ptau_181_ was higher than the unadjusted values (adjusted: *p* = 2.82 × 10^-26^, r = 0.573 vs. unadjusted: *p* = 8.98 × 10^-22^, r = 0.521; Table 2). We used the R package cocor [32] and determined that this change in correlation was statistically significant (*p* = 0.006, 95 % CI: -0.116, -0.020; Table 2). Similar results were found for tau (Table 2). As expected, we did not find any significant difference in the correlation between Aβ_42_ and the corrected or uncorrected YKL-40 values (Table 2). For CSF Tau/Aβ_42_ ratio, which is a powerful predictor for AD and progression, we found a marginally significant improvement when the genetic information was included (adjusted: r = 0.357 vs. unadjusted: *r* = 0.318; *p* = 0.071, 95 % CI: -0.083, 0.003, Table 2).

## Discussion

AD pathology is present long before any clinical symptoms [5, 21, 40–45], and multiple clinical trials are being performed in pre-symptomatic individuals. However, it is necessary to have reliable biomarkers to identify these individuals and to monitor the efficacy of new treatments. CSF ptau_181_ and Aβ_42_ have emerged as the most promising biochemical biomarkers for AD risk and progression [46]. Although these CSF biomarker levels are highly associated with AD, there is large inter-individual variability, and a relatively large overlap in the absolute CSF levels between cases and controls. Additionally, if treatments directly target tau proteins or Aβ_42_, the CSF levels may no longer be an informative biomarker because the treatment could affect those levels separately from disease state. Therefore, the identification of surrogates for Aβ_42_ and tau and novel approaches to improve accuracy of CSF biomarkers that can control for the individual inter-variability can have a large impact on clinical trials as well as in the general practice.

CSF YKL-40 has emerged as a novel potential biomarker for AD, as it is higher in individuals with AD than in cognitively normal individuals and is highly correlated with CSF ptau_181_ levels [2, 12, 13, 15]. However, it is not clear whether CSF YKL-40 is simply a biomarker or also an endophenotype for AD. Biomarkers are measurable biological characteristics that can be used as indicators of complex traits, but don’t necessarily provide information about the biology of the trait. They may simply be influenced by the same biological processes rather than being part of those processes. Endophenotypes are biomarkers that are heritable traits with a genetic connection with disease, can be measured in all individuals regardless of disease status, and therefore can be highly informative about the biological causes of the disease.

In this study, we used genomic approaches to determine whether CSF YKL-40 is an endophenotype for AD, and whether CSF YKL-40 becomes more informative by adding genetic information. Here we reported for the first time a genetic analysis for CSF YKL-40 levels. We found a genome-wide significant signal on *CHI3L1*, which encodes YKL-40 (rs10399931: *p* = 1.76 × 10^-14^, MAF = 0.24), and multiple suggestive signals on other chromosomes. Interestingly, rs10399931 alone explains almost as much of the variance in YKL-40 levels (R^2^ = 0.127) as both age and gender combined (R^2^ = 0.149). Neither this variant or any of the suggestive variants were associated with AD risk, age at onset, or disease progression, indicating that CSF YKL-40 is probably not an endophenotype for AD.

Because of the large proportion of CSF YKL-40 variability explained by rs10399931, we analyzed whether the correlation with CSF ptau_181_ improves when including the genotypic information for this variant. We hypothesize that in order to use genetic information to improve biomarker efficacy, the genetic association must be strong and replicable. We also predict that the improvement of the efficacy of the biomarker could be correlated with the proportion of biomarker level variability explained by the genetic variant. Additionally, if multiple loci are significantly associated with biomarker levels, a polygenic risk score should provide better performance than single locus analyses. As hypothesized, we found a significant increase in the correlation of CSF YKL-40 with tau and ptau_181_ when genetic information for rs10399931 was included in the model. The correlation with the Tau/Aβ_42_ ratio also improved, although it did not reach statistical significance. Based on these results, we hypothesize that the addition of genotypic information could significantly increase the sensitivity and specificity of biomarkers, but strong genetic associations are needed. When multiple loci are associated with biomarker levels, genetic risk scores would probably provide better results.

Additionally, we think this approach for improving biomarker efficacy by adjusting for genetic effect would only be effective for biomarkers, as may be the case for YKL-40, and not for informative endophenotypes such as CSF levels of Aβ_42_ and tau. Genetic variants associated with endophenotype values are very likely to be associated with disease risk as well, as has been found previously in the case of AD endophenotypes [18, 47]. Therefore, correcting for genetic variants associated with endophenotype levels would generate a co-linearity problem and not improve, but actually worsen, the biomarker performance in that case.

## Conclusions

Our genetic analyses indicate that although CSF YKL-40 levels are a promising biomarker for AD, there is no evidence they are an AD endophenotype. Genetic variation highly regulates CSF YKL-40 levels, and by including genetic information, the correlations with CSF tau and ptau_181_ levels increase. Although additional studies are needed to confirm our hypothesis, our results suggest that studies of potential biomarkers for complex traits may benefit from correction of genetic effects on biomarker levels. This could be particularly important when using biomarkers as surrogate endpoints in clinical trials.

## Additional files

Additional file 1: Table S1. Covariate associations with CSF YKL-40. Results (*p*-value and adjusted R^2^) from regression of potentially confounding covariates: age at lumbar puncture, gender, and sample batch. Only age appeared significantly associated with CSF levels of YKL-40 (*p* = 1.19 × 10^-18^, R^2^ = 0.184). (DOCX 16 kb)
Additional file 2: Supplementary Methods and Results. Methods and results for the gene ontology over-representation analyses of top SNPs from the single variant analysis of CSF YKL-40 levels. 70 genes mapped to the top SNPs (*p* < 1 × 10^-4^) were significantly enriched in human brain and pituitary tissue at all levels of specificity. Figure illustrates the different human tissues with expression data available with Benjamini-Hochberg corrected *p* < 0.10 in the enrichment analysis and the Table shows the results for all tissues with uncorrected *p* < 0.05 in at least one specificity index. The specificity index represents how specific a set of genes are to a particular tissue. (DOCX 163 kb)
Additional file 3: Figure S1. Beeswarm plot of the normalized CSF YKL-40 levels in cases (defined as CDR > 0 at time of lumbar puncture) compared to controls (CDR = 0 at time of lumbar puncture). *p* = 0.015. (DOCX 119 kb)
Additional file 4: Figure S2. Scatterplots of correlations between normalized values of CSF YKL-40 and Tau/Aβ_42_ ratio, levels of Aβ_42_, ptau_181_, and tau. Pearson’s correlation (r). CSF YKL-40 positively correlated with Tau/Aβ_42_ ratio (a), ptau_181_ (c), and tau (d), but was not correlated with Aβ_42_ (b). (DOCX 89 kb)
Additional file 5: Table S2. YKL-40 single variant analysis top loci (*p* < 1 × 10^-5^). Chr = chromosome, bp position = base pair position, SNP = rs ID for single nucleotide polymorphism, MAF = minor allele frequency (also effect allele), Gene = nearest gene. Chromosome and base pair position based on Build 37 of reference genome. (DOCX 14 kb)
Additional file 6: Table S3. Top gene ontology categories (*p* < 0.05) in both CPDB and PANTHER over-representation analyses of YKL-40 GWAS results (*p* < 1 × 10^-4^). GO ID = Identification number from the Gene Ontology Consortium, CPDB = Consensus Path Database, PANTHER = Protein Analysis Through Evolutionary Relationships. Thirteen gene ontology terms had *p* < 0.05 in both analyses. (DOCX 13 kb)

## Abbreviations

AD: Alzheimer’s disease
APOE: Apolipoprotein E
Aβ_42_: Amyloid beta (1-42)
CDR: Clinical dementia rating
CDR-SB: CDR Sum of Boxes
CHI3L1: Chitinase-3-like 1
CSF: Cerebrospinal fluid
ELISA: Enzyme-linked immunosorbent assay
eQTL: Expression quantitative trait locus
GWAS: Genome-wide association study
Knight-ADRC: Charles F. and Joanne Knight Alzheimer’s Disease Research Center
MAF: Minor allele frequency
ptau_181_: Phosphorylated tau (181)
SNP: Single nucleotide polymorphism
