# Supplementary material for: Chitinase-3-like 1 protein (CHI3L1) locus influences cerebrospinal fluid levels of YKL-40
Source: BMC Neurol. 2016 Nov 10;16:217. doi: 10.1186/s12883-016-0742-9 (PMC5105244; doi:10.1186/s12883-016-0742-9)
Supplement: Additional file 1: Table S1. — Covariate associations with CSF YKL-40. Results (p-value and adjusted R2) from regression of potentially confounding covariates: age at lumbar puncture, gender, and sample batch. Only age appeared significantly associated with CSF levels of YKL-40 (p = 1.19 × 10^-18^, R2 = 0.184). (DOCX 16 kb) [file 12883_2016_742_MOESM1_ESM.docx]

| Table S1. Covariate associations with CSF YKL-40. | | |
| --- | --- | --- |
|  | **CSF YKL-40** | |
| **Covariate** | **p-value** | **R^2^** |
| Age | **1.19×10^-18^** | **0.184** |
| Gender | 0.769 | -0.002 |
| Sample batch | 0.912 | 0.002 |
