# Supplementary material for: Chitinase-3-like 1 protein (CHI3L1) locus influences cerebrospinal fluid levels of YKL-40
Source: BMC Neurol. 2016 Nov 10;16:217. doi: 10.1186/s12883-016-0742-9 (PMC5105244; doi:10.1186/s12883-016-0742-9)
Supplement: Additional file 2: — Supplementary Methods and Results. Methods and results for the gene ontology over-representation analyses of top SNPs from the single variant analysis of CSF YKL-40 levels. 70 genes mapped to the top SNPs (p < 1 × 10^-4^) were significantly enriched in human brain and pituitary tissue at all levels of specificity. Figure illustrates the different human tissues with expression data available with Benjamini-Hochberg corrected p < 0.10 in the enrichment analysis and the Table shows the results for all tissues with uncorrected p < 0.05 in at least one specificity index. The specificity index represents how specific a set of genes are to a particular tissue. (DOCX 163 kb) [file 12883_2016_742_MOESM2_ESM.docx]

**Supplementary Methods and Results**

**Supplementary Methods**

*Gene ontology over-representation analyses*

GWAS SNPs were pruned using the clump function in PLINK v1.9 [1]. SNPs were clumped if they were located within 1Mb and in LD (r=0.8) with an index SNP, defined as the SNP with the lowest p-value within an LD region. Index SNPs with GWAS p<1×10^-4^ were mapped to genes classified as protein coding using a gene map created with the Table Browser tool on the UCSC genome browser using the Feb. 2009 (GRCh37/hg19) assembly (http://www.genome.ucsc.edu, accessed March 18, 2015) [2,3]. SNPs were mapped to a gene if they were located within 20kb of that gene and if SNPs mapped to more than one gene, all genes were included in the analysis. Genes were only counted once regardless of how many SNPs were mapped to the gene. There were 157 index SNPs in the YKL-40 single variant analysis with p<1×10^-4^ that mapped to 70 genes.

Gene ontology analyses were performed using the Protein Analysis Through Evolutionary Relationships (PANTHER) statistical over-representation test release 20150430 (http://www.go.pantherdb.org) [4,5] and the ConsensusPathDB (CPDB) over-representation gene set analysis release 30 (http://www.cpdb.molgen.mpg.de) [6]. PANTHER used data from the Gene Ontology Consortium (GOC) (http://www.geneontology.org) released August 6, 2015 and CPDB used GOC version GO_201501 released January 2015. PANTHER calculated over-representation of candidate genes, relative to the background, for various gene ontology (GO) terms using a binomial distribution test. The multiple test correction option used by PANTHER is the Bonferroni model which is highly conservative in this case because ontology terms include parent and child terms which are not independent but are all tested in the analysis, so we decided to use PANTHER without Bonferroni correction. We used the default background gene set for PANTHER which included 20,814 genes from the human gene database obtained from the European Bioinformatics Institute Reference Proteomes dataset release 2014_4. CPDB compared rates of GO term membership between the background and candidate gene sets using the hypergeometric test and the false discovery rate method of multiple test correction. We used the default background gene set for CPDB which included 18,043 genes with HUGO Gene Nomenclature Committee IDs that were annotated to at least one GO term. Of the 70 genes mapped from the YKL-40 GWAS results, 67 were used by PANTHER and 63 were used by CPDB. Categories with p<0.05 in both analyses were considered significant.

*Tissue specific expression analyses*

The genes mapped from pruned SNPs with GWAS p<1×10^-4^ were entered in the Tissue Specific Expression Analysis (TSEA) tool v1.0 updated 03/03/14 (http://genetics.wustl.edu/jdlab/tsea) [7]. TSEA used Fisher’s Exact test with Benjamini-Hochberg multiple test correction to calculate the overlap of our candidate gene lists with lists of genes enriched in various tissues. TSEA used RNA-Seq data from the Genotype-Tissue Expression project (GTEx Analysis Pilot Data, January 31, 2013) [8] to determine gene expression enriched in 25 types of tissue at different Specificity Index (SI) thresholds. The SI is a statistical method used to quantify the specificity of gene expression in different tissue types relative to the other tissue types; lower SI values represent expression that is more specific to a particular tissue type relative to other types [7]. Of the 70 genes mapped from the YKL-40 GWAS, 69 genes were in the tissue gene expression dataset.

**Supplementary Results**

*Gene enrichment analyses from GWAS results*

We selected SNPs with p<1×10^-4^ from our single variant analyses and mapped them to protein coding genes (70 genes). We found that the genes from the YKL-40 results were significantly enriched very specifically in the brain (Specificity Index (SI) 0.001: corrected p=0.038; Figure on page 3 and Table on page 4). After analyzing the genes associated with levels of YKL-40 using ConsensusPathDB (CPDB) and Protein Analysis Through Evolutionary Relationships (PANTHER) we found 13 gene ontology terms significantly enriched in both analyses (Supplementary File 6).

**References**

1. Chang CC, Chow CC, Tellier LC, Vattikuti S, Purcell SM, Lee JJ. Second-generation PLINK: rising to the challenge of larger and richer datasets. Gigascience. 2015;4:7.

2. Karolchik D, Hinrichs AS, Furey TS, Roskin KM, Sugnet CW, Haussler D, et al. The UCSC Table Browser data retrieval tool. Nucleic Acids Res. 2004;32:D493–6.

3. Kent WJ, Haussler D. Assembly of the working draft of the human genome with GigAssembler. Genome Res. 2001;11:1541–8.

4. Mi H, Muruganujan A, Thomas PD. PANTHER in 2013: modeling the evolution of gene function, and other gene attributes, in the context of phylogenetic trees. Nucleic Acids Res. 2013;41:D377–86.

5. Thomas PD, Kejariwal A, Guo N, Mi H, Campbell MJ, Muruganujan A, et al. Applications for protein sequence-function evolution data: mRNA/protein expression analysis and coding SNP scoring tools. Nucleic Acids Res. 2006;34:W645–50.

6. Kamburov A, Wierling C, Lehrach H, Herwig R. ConsensusPathDB--a database for integrating human functional interaction networks. Nucleic Acids Res. 2009;37:D623–8.

7. Dougherty JD, Schmidt EF, Nakajima M, Heintz N. Analytical approaches to RNA profiling data for the identification of genes enriched in specific cells. Nucleic Acids Res. 2010;38:4218–30.

8. GTEx Consortium. The Genotype-Tissue Expression (GTEx) project. Nat. Genet. 2013;45:580–5.


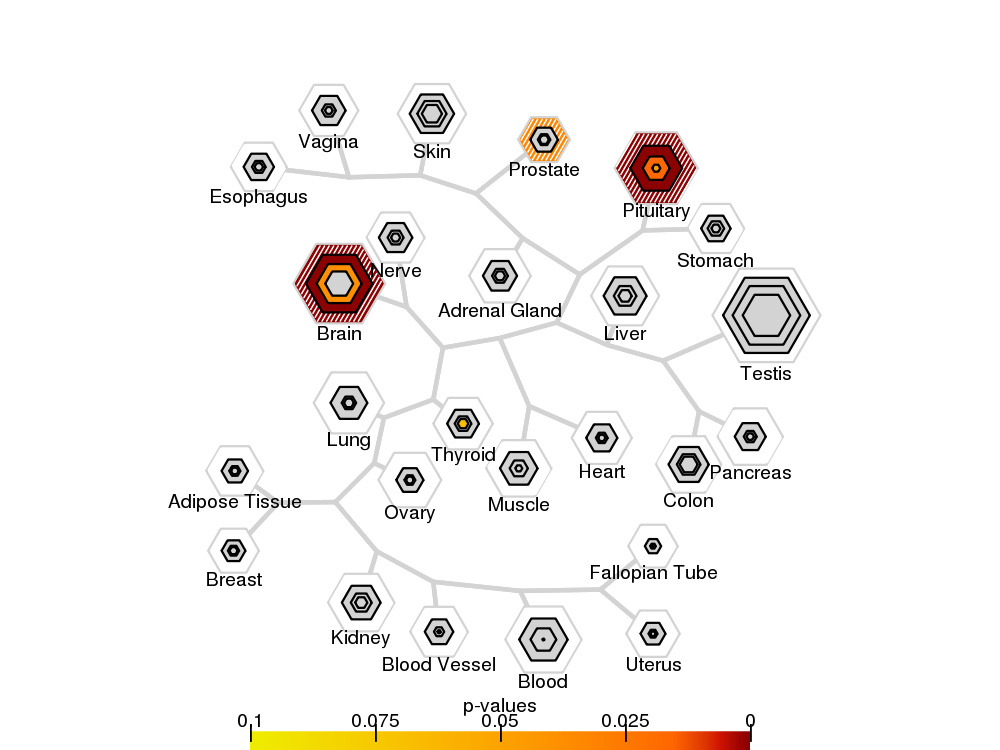


**Figure: Enrichment of CSF YKL-40 GWAS genes (mapped from SNPs with *P* < 1×10^-4^) expressed in different human tissue types.** Genes that are most specific to the particular tissue type relative to the other types are represented by the inner hexagons, growing less specific outwards. P-values are Benjamini-Hochberg corrected.

| **Table: Tissue specific expression analysis of YKL-40 GWAS results**. Fisher exact p-values with Benjamini-Hochberg corrected p-value in parentheses for each specificity index. Lower specificity index indicates more specific gene subset for that tissue. p<0.05 in bold. | | | | |
| --- | --- | --- | --- | --- |
|  | Specificity Index (SI) | | | |
| Tissue | 0.05 | 0.01 | 0.001 | 0.0001 |
| Brain | **3.74×10^-7^ (9.34×10^-6^)** | **5.31×10^-7^ (1.33×10^-5^)** | **0.003 (0.038)** | **0.038 (0.318)** |
| Fallopian Tube | 0.2 (0.537) | 0.227 (0.944) | **0.034 (0.212)** | 1.0 (1.0) |
| Muscle | 0.375 (0.672) | **0.046 (0.381)** | 1.0 (1.0) | 1.0 (1.0) |
| Pituitary | **8.28×10^-7^ (1.04×10^-5^)** | **7.37×10^-6^ (9.21×10^-5^)** | **6.41×10^-4^ (0.016)** | **0.002 (0.047)** |
| Prostate | **0.004 (0.031)** | 1.0 (1.0) | 1.0 (1.0) | 1.0 (1.0) |
| Thyroid | **0.049 (0.267)** | 0.073 (0.454) | **0.029 (0.212)** | **0.005 (0.068)** |
