# Supplementary material for: Chitinase-3-like 1 protein (CHI3L1) locus influences cerebrospinal fluid levels of YKL-40
Source: BMC Neurol. 2016 Nov 10;16:217. doi: 10.1186/s12883-016-0742-9 (PMC5105244; doi:10.1186/s12883-016-0742-9)
Supplement: Additional file 3: Figure S1. — Beeswarm plot of the normalized CSF YKL-40 levels in cases (defined as CDR > 0 at time of lumbar puncture) compared to controls (CDR = 0 at time of lumbar puncture). p = 0.015. (DOCX 119 kb) [file 12883_2016_742_MOESM3_ESM.docx]

**
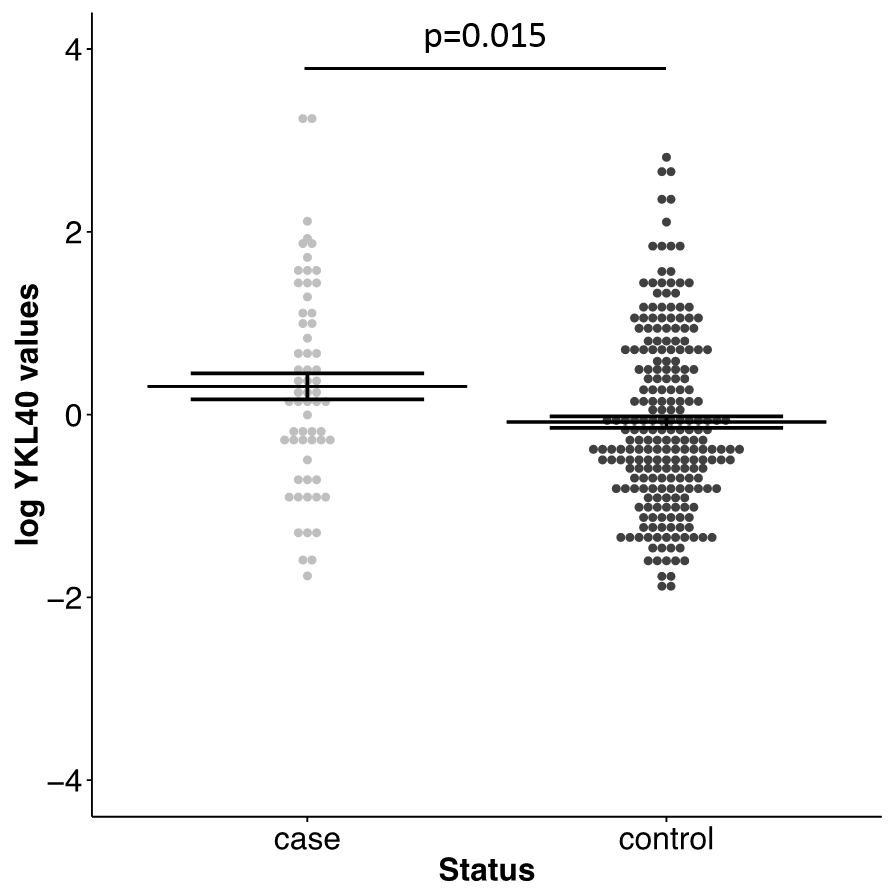
**

**Figure S1.** Log transformed levels of CSF YKL-40 by AD status (cases defined as CDR>0) at time of lumbar puncture. Error bars are based on s.e.m.
