# Supplementary material for: Chitinase-3-like 1 protein (CHI3L1) locus influences cerebrospinal fluid levels of YKL-40
Source: BMC Neurol. 2016 Nov 10;16:217. doi: 10.1186/s12883-016-0742-9 (PMC5105244; doi:10.1186/s12883-016-0742-9)
Supplement: Additional file 4: Figure S2. — Scatterplots of correlations between normalized values of CSF YKL-40 and Tau/Aβ42 ratio, levels of Aβ42, ptau181, and tau. Pearson’s correlation (r). CSF YKL-40 positively correlated with Tau/Aβ42 ratio (a), ptau181 (c), and tau (d), but was not correlated with Aβ42 (b). (DOCX 89 kb) [file 12883_2016_742_MOESM4_ESM.docx]

**Figure S2.** Scatterplots of correlations between normalized values of CSF YKL-40 and Tau/Aβ_42_ ratio, levels of Aβ_42_, ptau, and tau. Pearson’s correlation (r) **A)** YKL-40 vs Tau/Aβ_42_ ratio; **B)** YKL-40 vs Aβ_42_; **C)** YKL-40 vs ptau_181_; **D)** YKL-40 vs tau.
