# Supplementary material for: Chitinase-3-like 1 protein (CHI3L1) locus influences cerebrospinal fluid levels of YKL-40
Source: BMC Neurol. 2016 Nov 10;16:217. doi: 10.1186/s12883-016-0742-9 (PMC5105244; doi:10.1186/s12883-016-0742-9)
Supplement: Additional file 5: Table S2. — YKL-40 single variant analysis top loci (p < 1 × 10^-5^). Chr = chromosome, bp position = base pair position, SNP = rs ID for single nucleotide polymorphism, MAF = minor allele frequency (also effect allele), Gene = nearest gene. Chromosome and base pair position based on Build 37 of reference genome. (DOCX 14 kb) [file 12883_2016_742_MOESM5_ESM.docx]

| Table S2. YKL-40 single variant analysis top loci (*p* < 1×10^-5^). | | | | | | | | |
| --- | --- | --- | --- | --- | --- | --- | --- | --- |
|  |  |  |  |  |  |  |  | **RegulomeDB** |
| **Chr** | **bp position** | **SNP** | **MAF** | ***p*** | **beta** | **Gene** | **Function class** | **score** |
| 1 | 203156080 | rs10399931 | 0.244 | 1.76×10^-14^ | -0.575 | *CHI3L1* | upstream | 1f |
| 13 | 70574148 | rs78081700 | 0.074 | 6.26×10^-8^ | 0.636 | *KLHL1* | intronic | 7 |
| 1 | 111065073 | rs7537928 | 0.126 | 6.29×10^-7^ | 0.434 | *KCNA10(dist=3276)* | intergenic | 7 |
| 2 | 58997712 | rs10210385 | 0.386 | 1.03×10^-6^ | 0.314 | *LINC01122* | ncRNA_intronic | 6 |
| 20 | 3252348 | rs1922990 | 0.213 | 1.93×10^-6^ | 0.378 | *C20orf194* | intronic | 7 |
| 9 | 13530295 | rs933035 | 0.467 | 2.39×10^-6^ | -0.486 | *FLJ41200(dist=98967)* | intergenic | 6 |
| 21 | 42938352 | rs2838067 | 0.282 | 2.50×10^-6^ | -0.327 | *TMPRSS2(dist=58267)* | intergenic | 5 |
| 1 | 203159953 | rs10800904 | 0.403 | 2.72×10^-6^ | -0.294 | *CHI3L1(dist=4031)* | intergenic | 5 |
| 15 | 80318399 | rs7165414 | 0.310 | 2.94×10^-6^ | -0.334 | *ZFAND6(dist=33511)* | intergenic | 7 |
| 13 | 70579089 | rs57227774 | 0.095 | 3.40×10^-6^ | 0.505 | *KLHL1* | intronic | 6 |
| 4 | 181323550 | rs12641056 | 0.062 | 3.82×10^-6^ | -0.627 | *LINC00290(dist=661693)* | intergenic | 7 |
| 1 | 11029478 | rs12085319 | 0.235 | 4.40×10^-6^ | 0.568 | *C1orf127* | intronic | 2b |
| 13 | 89608237 | rs61961631 | 0.100 | 8.69×10^-6^ | -0.496 | *LINC00440(dist=279308)* | intergenic | 7 |
| 3 | 116008353 | rs6438312 | 0.153 | 9.06×10^-6^ | 0.403 | *LSAMP* | intronic | 6 |
| 15 | 80323896 | rs11630958 | 0.241 | 9.79×10^-6^ | -0.365 | *ZFAND6(dist=28014)* | intergenic | 5 |
