# Supplementary material for: Chitinase-3-like 1 protein (CHI3L1) locus influences cerebrospinal fluid levels of YKL-40
Source: BMC Neurol. 2016 Nov 10;16:217. doi: 10.1186/s12883-016-0742-9 (PMC5105244; doi:10.1186/s12883-016-0742-9)
Supplement: Additional file 6: Table S3. — Top gene ontology categories (p < 0.05) in both CPDB and PANTHER over-representation analyses of YKL-40 GWAS results (p < 1 × 10^-4^). GO ID = Identification number from the Gene Ontology Consortium, CPDB = Consensus Path Database, PANTHER = Protein Analysis Through Evolutionary Relationships. Thirteen gene ontology terms had p < 0.05 in both analyses. (DOCX 13 kb) [file 12883_2016_742_MOESM6_ESM.docx]

| **Table S3. Top gene ontology categories (p<0.05) in both CPDB and PANTHER over-representation analyses of YKL-40 GWAS results.** | | | |
| --- | --- | --- | --- |
| **GO ID** | **Term name** | **CPDB *p*** | **PANTHER *p*** |
| GO:0017080 | sodium channel regulator activity | 2.09×10^-3^ | 1.77×10^-4^ |
| GO:0097458 | neuron part | 1.54×10^-2^ | 3.52×10^-4^ |
| GO:0036477 | somatodendritic compartment | 1.75×10^-2^ | 1.43×10^-4^ |
| GO:0030425 | dendrite | 1.96×10^-2^ | 3.94×10^-4^ |
| GO:0034703 | cation channel complex | 2.27×10^-2^ | 2.12×10^-3^ |
| GO:1990351 | transporter complex | 2.99×10^-2^ | 3.92×10^-3^ |
| GO:0043197 | dendritic spine | 2.99×10^-2^ | 4.71×10^-3^ |
| GO:0043005 | neuron projection | 3.03×10^-2^ | 2.28×10^-3^ |
| GO:0043025 | neuronal cell body | 3.28×10^-2^ | 1.71×10^-3^ |
| GO:0031683 | G-protein beta/gamma-subunit complex binding | 3.42×10^-2^ | 2.36×10^-3^ |
| GO:0044309 | neuron spine | 3.47×10^-2^ | 4.96×10^-3^ |
| GO:0034702 | ion channel complex | 3.56×10^-2^ | 2.16×10^-3^ |
| GO:1902495 | transmembrane transporter complex | 3.95×10^-2^ | 3.62×10^-3^ |
